# SBMLToolkit.jl: a Julia package for importing SBML into the SciML ecosystem

**DOI:** 10.1515/jib-2024-0003

**Published:** 2024-05-28

**Authors:** Paul F. Lang, Anand Jain, Christopher Rackauckas

**Affiliations:** Deep Origin, South San Francisco, USA; JuliaHub, Boston, USA; JuliaHub, Boston, USA; Computer Science and Artificial Intelligence Laboratory (CSAIL), Massachusetts Institute of Technology, Boston, USA

**Keywords:** systems biology markup language, SBML, Julia, scientific machine learning

## Abstract

Julia is a general purpose programming language that was designed for simplifying and accelerating numerical analysis and computational science. In particular the Scientific Machine Learning (SciML) ecosystem of Julia packages includes frameworks for high-performance symbolic-numeric computations. It allows users to automatically enhance high-level descriptions of their models with symbolic preprocessing and automatic sparsification and parallelization of computations. This enables performant solution of differential equations, efficient parameter estimation and methodologies for automated model discovery with neural differential equations and sparse identification of nonlinear dynamics. To give the systems biology community easy access to SciML, we developed SBMLToolkit.jl. SBMLToolkit.jl imports dynamic SBML models into the SciML ecosystem to accelerate model simulation and fitting of kinetic parameters. By providing computational systems biologists with easy access to the open-source Julia ecosystevnm, we hope to catalyze the development of further Julia tools in this domain and the growth of the Julia bioscience community. SBMLToolkit.jl is freely available under the MIT license. The source code is available at https://github.com/SciML/SBMLToolkit.jl.

## 1 Introduction

The Systems Biology Markup Language (SBML) [1] is a standardized format to represent, store and exchange mathematical models of biochemical processes. There are currently over 3000 models on the BioModels repository [2, 3] that can be downloaded and used with SBML-compliant software. SBML is often compared to another systems biology format called CellML [4], which is widely adopted in physiological models. Both formats are encoded in XML. However, in contrast to the math-centric CellML format, SBML uses a reaction-centric approach. Key elements of typical SBML files are a listOfSpecies, detailing initial conditions or initial assignments, a listOfParameters, and a listOfReactions, comprising a listOfReactants, a listOfProducts and a kineticLaw. SBML offers extensive flexibility, allowing users to define complicated mathematical expressions in the listOfFunctions and to specify events in the listOfEvents. Each event consist of a trigger and at least one eventAssignment that sets a variable to a desired value or expression. Additionally, users can define assignmentRules (one variable is assigned to a value), algebraicRules (constraint equations that must evaluate to zero at all times) and rateRules (a differential equation) in the listOfRules. To avoid potential conflicts between rules and reactions, SBML introduces the boundaryCondition attribute on species. If boundaryCondition is true, rules override reactions, otherwise the species can only appear either in rules or reactions [5]. By emphasizing biochemical descriptions over mathematical equations, SBML enables researchers to define biochemical mechanisms independently of the simulation algorithm. SBML is supported by numerous tools in various established programming languages, including Python (e.g. via Tellurium [6, 7] and Antimony [8, 9]), Matlab (e.g. via SBMLToolbox [10] and Systems Biology Toolbox [11]) and R (e.g. via SBMLR [12]). Additionally, Copasi serves as a standalone software with a graphical user interface and extensive SBML support [13]. A more complete and up-to-date enumeration of SBML-compliant tools can be found on the SBML website.

**Figure 1:**
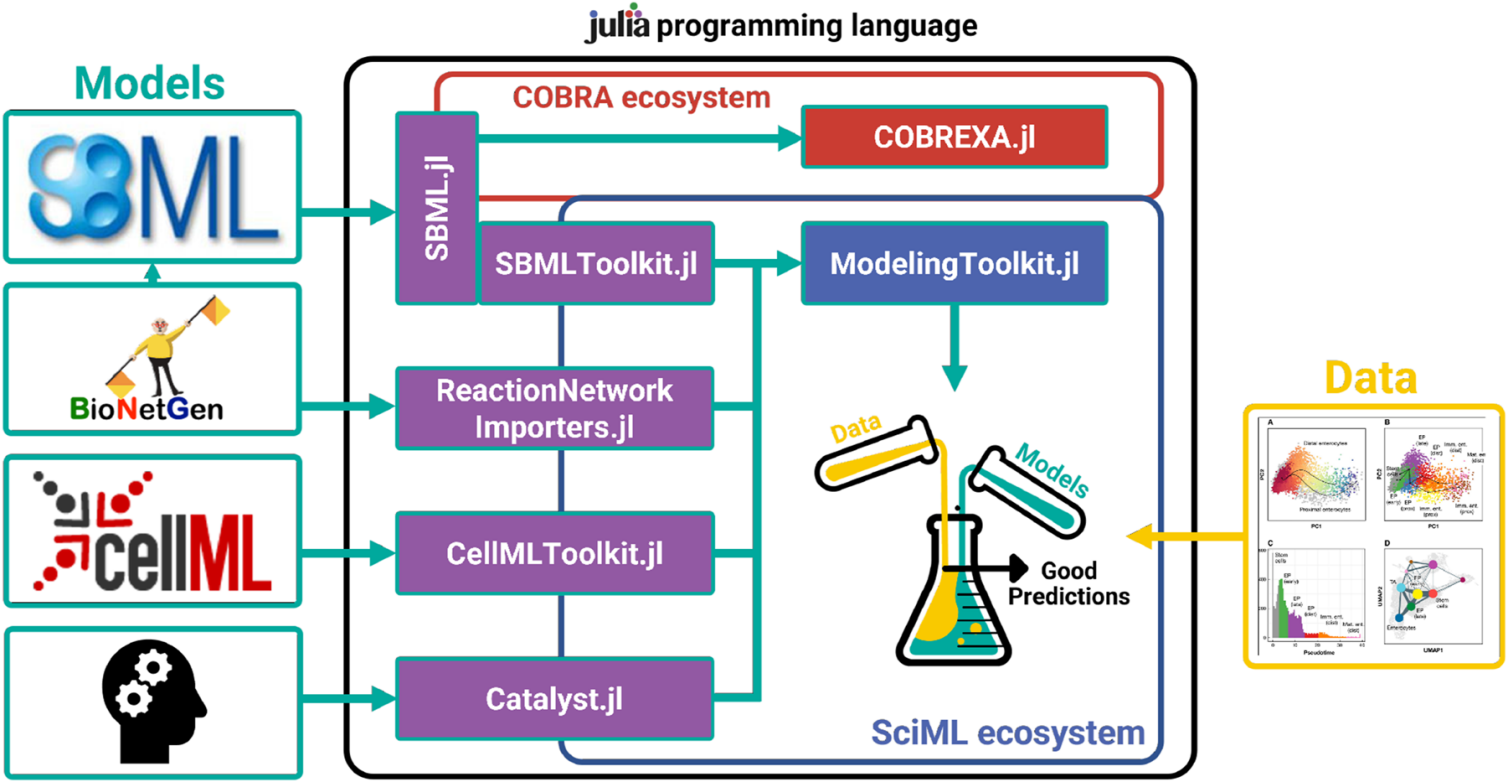
SBMLToolkit.jl connects systems biology formats like SBML to the Julia ecosystem for scientific machine learning. Figure adapted from [20].

More recently, the Julia programming language [14] has also gained popularity in scientific computing. Designed to bridge the gap between the high-level and easy-to-use languages like Python, and the computational speed of low-level, compiled languages such as C and Fortran, Julia has become a valuable asset in accelerating research from ideation to code development [15]. The high-level, yet fast code is in large part enabled by multiple dispatch. Multiple dispatch allows to create multiple functions/methods with the same name, each of which specializes on certain input types. This facilitates writing type-stable functions that enable the compiler to generate efficient code. The Julia package DifferentialEquations.jl is particularly relevant for systems biologists, as it provides access to cutting-edge solvers that perform very well in benchmarks [16, 17]. Additionally Julia has a strong ecosystem for Scientific Machine Learning (SciML), where researchers can integrate mechanistic models with interpretable machine learning models to discover previously unknown biological mechanisms [18, 19]. To provide the systems biology community with access to the Julia SciML ecosystem and its extensive array of fast solvers within DifferentialEquations.jl, we developed SBMLToolkit.jl, an importer for dynamic SBML models into the SciML ecosystem (Figure 1).

## 2 Implementation

SBMLToolkit.jl imports SBML models via the SBML.jl package [21], which provides the Model type – a Julia type that closely resembles the anatomy of SBML models. SBML.jl relies on the SBML_jll.jl binary wrapper of the libSBML C library [22]. SBMLToolkit.jl, was designed to comply with Level 3 version 2 of the SBML format. The set_level_and_version function provides the user with an easy interface to convert to this level and version. Additionally, the user has fine control on how to process the SBML file during import via the libsbml_convert function. When such fine control is not needed, the user can simply call convert_simplify_math to expand functions and initialAssignments and promote localParameters. However, the flattening/hard-coding of SBML initialAssignments removes the dependency of the simulation results of parameters in the initialAssignment. Especially for parameter estimation, we therefore recommend using convert_promotelocals_expandfuns as alternative.

In SBMLToolkit.jl, SBML.jl Models are always converted to Catalyst.jl ReactionSystems [23] via a series of steps. First, SBMLToolkit parses the reactions. During this step SBMLToolkit.jl tries to split any bidirectional reaction into a forward and reverse part. This separation does not affect deterministic simulations, but is required for accurate simulation with stochastic simulation algorithms. Second, SBMLToolkit.jl parses compartments, parameter values, initial conditions of species and initialAssigments. SBML compartment volumes, species and parameters are interpreted as Catalyst.jl parameters if they are not time-varying, and as species otherwise. If initialAssignments exist, they override initial conditions. Third, assignmentRules, algebraicRules and rateRules are parsed. For assignmentRules and rateRules, this again involves overriding initial conditions. Next, SBMLToolkit.jl parses events. However, event support is currently incomplete. For example, event triggers are specified with Boolean expressions in SBML, and with numeric expressions in Catalyst.jl (or more specifically in ModelingToolkit.jl [24], a symbolic-numeric computation package Catalyst.jl depends upon). In SBML events are triggered when the Boolean expression (e.g. *Vol*(*t*) ≥ 2 ⋅ *Vol*_init_) transitions from false to true. In ModelingToolkit.jl events are triggered when the numeric expression (e.g. *Vol*(*t*) − 2 ⋅ *Vol*_init_) evaluates to zero. As there is currently no easy way to restrict the trigger to either up or downpass of the zero threshold in ModelingToolkit.jl, SBMLToolkit.jl currently triggers events regardless of directionality (which empirically aligns better with the user’s intention than not triggering events at all). To prevent unexpected simulation outcomes, SBMLToolkit.jl alerts the user with a warning whenever they attempt to import SBML models containing events. Finally, all the information gathered from the SBML file is synthesized into a Catalyst.jl ReactionSystem, making sure that volumes, and combinations of SBML Species attributes like boundaryCondition, constant and hasOnlySubstanceUnits are handled correctly. It is important to note that SBMLToolkit.jl currently treats species as absolute quantities rather than concentrations.

## 3 Usage and documentation

Prior to importing an SBML file, users are advised to employ SBMLToolkit’s

checksupport_file(“my_model.xml”)

to check if all features in the SBML file are supported. Unsupported features include SBML constraints, delays, and expressions containing factorials. Following a successful check, users can import the SBML file as an SBML.jl Model and specify the desired version and level using the set_level_and_version function. Preprocessing options such as promoting parameters that are local to certain reactions to the global namespace of the model, and expanding/flattening mathematical expressions from the listOfFunctions into all their occurrences can also be selected during import. For example, an SBML file called my_model.xml can be imported as an SBML.jl Model via

mdl = readSBML(“my_model.xml”, doc -> begin

set_level_and_version(3, 2)(doc)

convert_promotelocals_expandfuns(doc)

end)

Such a Model can then be converted to a Catalyst.jl ReactionSystem via

rs = ReactionSystem(mdl).

If the user wants to run a deterministic simulation, the ReactionSystem can be converted to a ModelingToolkit.jl ODESystem [24] via

odesys = convert(ODESystem, rs). Most users, however, will not need to control the internals of the import process. Therefore, we provide simple, single-line functions to create

– SBML.jl Models: mdl = readSBML(“my_model.xml”, DefaultImporter())
– Catalyst.jl ReactionSystems: rs = readSBML(“my_model.xml”, ReactionSystemImporter())
– ModelingToolkit.jl ODESystems: odesys = readSBML(“my_model.xml”, ODESystemImporter())

directly from an SBML file and without having to run checksupport_file(“my_model.xml”). Very often, however, models were optimized for human readability instead of numerical simulation. For ODESystems, we therefore strongly recommend using

odesys = structural_simplify(odesys),

which accelerates simulations, for instance by removing redundancies in the equations. Once imported, users gain access to the full capabilities of the SciML ecosystem [23]. A large variety of solvers for CPU [16] and GPU [25] can be employed to simulate the model. Parameter estimation and Bayesian approaches are facilitated by packages like DiffEqParamEstim.jl, Optimization.jl, and Turing.jl [18, 26]. Users who want to check for structural identifiability can do so via StructuralIdentifiability.jl. If the goodness of fit is insufficient, users can try to extend ReactionSystems or ODESystems with neural differential equations to approximate missing biology and potentially discover new mechanisms [18, 19]. Steady state analysis is supported by NonlinearSolve.jl or HomotopyContinuation.jl [27], and bifurcation analysis can be performed with BifurcationKit.jl [28]. Additionally, users can employ GraphViz to visualize the chemical reaction network and Latexify.jl to generate LaTeX equations [23].

## 4 Discussion

In summary, SBMLToolkit.jl is an open-source package that offers SBML users in the systems biology community a user-friendly and customizable gateway to the Julia SciML ecosystem. Despite its utility, several features remain unsupported, including directionality discrimination in event triggers, delays in events and equations, and automated conversion from amounts to concentrations. Additionally, the need for SBML export may arise as Catalyst.jl evolves to a popular domain-specific language for creating models of biochemical reaction systems. As the Julia community in systems biology grows, it is anticipated that these features will be addressed with increasing demand.

## References

[1] Hucka M, Finney A, Sauro HM, Bolouri H, Doyle JC, Kitano H (2003). The systems biology markup language (SBML): a medium for representation and exchange of biochemical network models. Bioinformatics.

[2] Glont M, Nguyen T, Graesslin M, Hälke R, Ali R, Schramm J (2018). BioModels: expanding horizons to include more modelling approaches and formats. Nucleic Acids Res.

[3] Malik-Sheriff RS, Glont M, Nguyen TVN, Tiwari K, Roberts MG, Xavier A (2020). BioModels—15 years of sharing computational models in life science. Nucleic Acids Res.

[4] Cuellar AA, Lloyd CM, Nielsen PF, Bullivant DP, Nickerson DP, Hunter PJ (2003). An overview of CellML 1.1, a biological model description language. Simulation.

[5] Hucka M, Bergmann F, Chaouiya C, Dräger A, Hoops S, Keating SM (2019). The systems biology markup language (SBML): language specification for level 3 version 2 core release 2. J Integr Bioinform.

[6] Medley JK, Choi K, König M, Smith L, Gu S, Hellerstein J (2018). Tellurium notebooks—an environment for reproducible dynamical modeling in systems biology. PLoS Comput Biol.

[7] Choi K, Medley JK, König M, Stocking K, Smith L, Gu S (2018). Tellurium: an extensible python-based modeling environment for systems and synthetic biology. Biosystems.

[8] Smith LP, Bergmann FT, Chandran D, Sauro HM (2009). Antimony: a modular model definition language. Bioinformatics.

[9] Jardine BE, Smith LP, Sauro HM (2023). MakeSBML: a tool for converting between Antimony and SBML. ..

[10] Keating SM, Bornstein BJ, Finney A, Hucka M (2006). SBMLToolbox: an SBML toolbox for MATLAB users. Bioinformatics.

[11] Schmidt H, Jirstrand M (2006). Systems Biology Toolbox for MATLAB: a computational platform for research in systems biology. Bioinformatics.

[12] Radivoyevitch T, Venkateswaran V (2023). SBMLR.

[13] Hoops S, Sahle S, Gauges R, Lee C, Pahle J, Simus N (2006). COPASI–a COmplex PAthway SImulator. Bioinformatics.

[14] Bezanson J, Edelman A, Karpinski S, Shah VB (2017). Julia: a fresh approach to numerical computing. SIAM Rev.

[15] Roesch E, Greener JG, MacLean AL, Nassar H, Rackauckas C, Holy TE (2023). Julia for biologists. Nat Methods.

[16] Rackauckas C, Nie Q (2017). DifferentialEquations.jl – a performant and feature-rich ecosystem for solving differential equations in Julia. J Open Res Software.

[17] Rackauckas C, Nie Q (2019). Confederated modular differential equation APIs for accelerated algorithm development and benchmarking. Adv Eng Software.

[18] Rackauckas C, Ma Y, Martensen J, Warner C, Zubov K, Supekar R (2020). Universal differential equations for scientific machine learning. ..

[19] Brunton SL, Proctor JL, Kutz JN (2016). Discovering governing equations from data by sparse identification of nonlinear dynamical systems. Proc Natl Acad Sci USA.

[20] Lang P (2022). Improving our mechanistic understanding of cell cycle dynamics.

[21] Kratochvíl M, Heirendt L, Wilken SE, Pusa T, Arreckx S, Noronha A (2022). COBREXA.jl: constraint-based reconstruction and exascale analysis. Bioinformatics.

[22] Bornstein BJ, Keating SM, Jouraku A, Hucka M (2008). LibSBML: an API library for SBML. Bioinformatics.

[23] Loman TE, Ma Y, Ilin V, Gowda S, Korsbo N, Yewale N (2023). Catalyst: fast and flexible modeling of reaction networks. PLoS Comput Biol.

[24] Ma Y, Gowda S, Anantharaman R, Laughman C, Shah V, Rackauckas C (2021). ModelingToolkit: a composable graph transformation system for equation-based modeling. ..

[25] Utkarsh U, Churavy V, Ma Y, Besard T, Srisuma P, Gymnich T (2024). Automated translation and accelerated solving of differential equations on multiple GPU platforms. Comput Methods Appl Mech Eng.

[26] Ge H, Xu K, Ghahramani Z (2018). Turing: a language for flexible probabilistic inference. Proceedings of the twenty-first international conference on artificial intelligence and statistics.

[27] Breiding P, Timme S (2018). HomotopyContinuation.jl: a package for homotopy continuation in Julia. ..

[28] Veltz R (2020). BifurcationKit.jl.

